# Disentangling effects of air and soil temperature on C allocation in cold environments: A ^14^C pulse‐labelling study with two plant species

**DOI:** 10.1002/ece3.4215

**Published:** 2018-07-13

**Authors:** Adele Ferrari, Frank Hagedorn, Pascal Alex Niklaus

**Affiliations:** ^1^ Department of Evolutionary Biology and Environmental Studies University of Zurich Zurich Switzerland; ^2^ Department of Forest Soils and Biogeochemistry Swiss Federal Institute for Forest, Snow and Landscape Research WSL Birmensdorf Switzerland; ^3^ University of Zurich Research Priority Program Global Change and Biodiversity University of Zurich Zurich Switzerland

**Keywords:** air‐soil temperature interaction, growth, *Leucanthemopsis alpina*, photosynthesis, *Pinus mugo*, soil and root respiration

## Abstract

Carbon cycling responses of ecosystems to global warming will likely be stronger in cold ecosystems where many processes are temperature‐limited. Predicting these effects is difficult because air and soil temperatures will not change in concert, and will affect above and belowground processes differently. We disentangled above and belowground temperature effects on plant C allocation and deposition of plant C in soils by independently manipulating air and soil temperatures in microcosms planted with either *Leucanthemopsis alpina* or *Pinus mugo* seedlings. Daily average temperatures of 4 or 9°C were applied to shoots and independently to roots, and plants pulse‐labelled with ^14^ CO_2_. We traced soil CO_2_ and ^14^ CO_2_ evolution for 4 days, after which microcosms were destructively harvested and ^14^C quantified in plant and soil fractions. In microcosms with *L. alpina*, net ^14^C uptake was higher at 9°C than at 4°C soil temperature, and this difference was independent of air temperature. In warmer soils, more C was allocated to roots at greater soil depth, with no effect of air temperature. In *P. mugo* microcosms, assimilate partitioning to roots increased with air temperature, but only when soils were at 9°C. Higher soil temperatures also increased the mean soil depth at which ^14^C was allocated. Our findings highlight the dependence of C uptake, use, and partitioning on both air and soil temperature, with the latter being relatively more important. The strong temperature‐sensitivity of C assimilate use in the roots and rhizosphere supports the hypothesis that cold limitation on C uptake is primarily mediated by reduced sink strength in the roots. We conclude that variations in soil rather than air temperature are going to drive plant responses to warming in cold environments, with potentially large changes in C cycling due to enhanced transfer of plant‐derived C to soils.

## 1 INTRODUCTION

In terrestrial ecosystems, most carbon (C) cycling processes are temperature‐sensitive, with lower rates observed at low temperature. Conversely, warming due to climate change is expected to accelerate C cycling, and these changes likely will be particularly large in cold ecosystem because these are most temperature‐limited in the first place (IPCC, 2013). The temperature sensitivity of isolated processes such as photosynthesis and respiration are relatively well studied (e.g., Davidson & Janssens, 2006; Yamori, Hikosaka, & Way, 2014). How these individual responses combine in complex natural ecosystems under realistic scenarios is less well understood, and the ultimate consequences of climate warming for future C cycling and ecosystem functioning therefore remain difficult to predict (Chapin et al., 2009).

Using meta‐analysis, Rustad, Campbell, Marion, and Norby (2001) compiled data from 20 studies that spanned a wide latitudinal and climatic range and found that, on average, biomass production increased by 19% under warming, with the largest values in cold ecosystems. Together with productivity, soil respiration and N mineralization also increased in many studies. The authors argued that plant productivity responses may have arisen from increased photosynthetic rates, longer growing seasons in studies with year‐round warming, and from improved plant N supply due to higher soil microbial activity and mineralization rates. More recent field experiments in alpine and artic conditions corroborate positive effects of warming on plant productivity (Dawes, Philipson, Fonti, & Bebi, 2015; Hudson, Henry, & Cornwell, 2011; Natali, Schuur, & Rubin, 2012; Sistla et al., 2013). For example, Sistla et al. (2013) found a 50% increase in aboveground vascular plant biomass in Alaskan tussock tundra after 14 years of experimental warming.

Soil microbial respiration exhibits a strong temperature dependency under controlled laboratory conditions (Kirschbaum, 1995). This temperature sensitivity is particularly large at low temperatures, suggesting the possibility of large ecosystem‐level C losses from cold ecosystem in which soil organic matter turns over only slowly. Warming thus may accelerate both primary production and decomposition, so that the ecosystem‐level consequences of these changes will depend on their relative magnitude (Crowther et al., 2016; Kirschbaum, 2000).

The above and belowground C cycle are closely coupled but they may experience different temperature regimes. While aboveground air and belowground soil temperatures are generally related, the degree of coupling depends on many factors including soil moisture (Ochsner, Horton, & Ren, 2001), insulation by snowpack (Maurer & Bowling, 2014), and the ratio of radiative to convective heat fluxes and their modifications by vegetation (Körner, 1998). Air and soil temperature are therefore expected to change differently with climate change (Jungqvist, Oni, Teutschbein, & Futter, 2014; Zhang, Chen, Smith, Riseborough, & Cihlar, 2005). It is therefore often unclear how future temperature regimes should most realistically be simulated (Hoch, 2013; Pumpanen, Heinonsalo, Rasilo, Villemot, & Ilvesniemi, 2012). Open‐top chambers are frequently used to passively warm ecosystems, however, they typically generate larger effects on air temperature than on soil temperature (Hobbie & Chapin, 1998; Hollister & Webber, 2006). In contrast, active warming systems such as buried heating cables predominantly warm soils (Hagedorn et al., 2010; Peterjohn, Melillo, & Steudler, 1994; Rustad & Fernandez, 1998). Overhead infrared lamps directly warm soil and canopy, with air warmed only indirectly (Kimball et al., 2008; Luo et al., 2010). Irrespective of the warming technique adopted, such studies will not allow to unequivocally separate effects of air and soil temperature unless these temperatures are manipulated independently.

Given that air and soil temperature do not change synchronously, a central question is whether air or soil temperature is more important in controlling plant productivity and C allocation. Leaves and the photosynthetic apparatus are affected directly by air temperature. Indeed, CO_2_ assimilation can increase because of a direct stimulation of photosynthesis in warmer air (Berry & Bjorkman, 1980; Medlyn et al., 2002; Yamori et al., 2014). On the other hand, photosynthetic rates also are controlled by the activity of sinks for assimilates (Iglesias, Lliso, Tadeo, & Talon, 2002; Pammenter, Loreto, & Sharkey, 1993; Repo, Leinonen, Ryyppö, & Finér, 2004; Savitch, Gray, & Huner, 1997; Turnbull, Murthy, & Griffin, 2002). A high assimilate consumption rate often induces an up‐regulation of photosynthesis, whereas inactive sinks cause a down‐regulation which often is paralleled by an accumulation of surplus carbohydrates in leaves. It has been posited that, in cold environments, plant growth may be more temperature‐limited than photosynthesis per se (Grace, 2002; Hoch & Körner, 2009; Hoch, Popp, & Körner, 2002). Low soil temperature may be a particularly critical determinant of this effect because strongly reduced root growth has been found below approximately 6–7°C (Alvarez‐Uria & Körner, 2007; Schenker, Lenz, Körner, & Hoch, 2014). In cold environments, low rates of photosynthesis may thus be the result of a low belowground sink activity in the soil rather than of low air temperatures.

In this study, we aimed at disentangling effects of air and soil temperature on CO_2_ uptake, allocation of assimilates within plants, and the fate of rhizodeposits in soils. We used experimental microcosms to expose a herbaceous and a woody plant species naturally found at the alpine treeline to temperature treatments. We factorially combined air and soil temperature treatments (levels: 4 and 9°C); this temperature range was chosen because it reflects a range that frequently occurs in the native habitat of the investigated species. We pulse‐labelled plants with ^14^CO_2_ and followed the fate of labelled assimilates through the plant‐soil system using liquid scintillation counting and an autoradiographic technique that allows to map the small‐scale belowground distribution of ^14^C (Hagedorn, Bruderhofer, Ferrari, & Niklaus, 2015; Rime & Niklaus, 2017; Stiehl‐Braun, Powlson, Poulton, & Niklaus, 2011). We focused on the short‐term (~1 week) consequences of both acclimation to temperature and assimilate fate after labelling because we were interested in the relatively immediate mechanisms that govern C allocation. Specifically, we hypothesized that belowground temperature would be a more important determinant of C allocation than aboveground temperature.

## 2 METHODS

We independently manipulated air and soil temperature of microcosms containing seedlings of either *Leucanthemopsis alpina* (L., Heywood) or *Pinus mugo* (Turra). We selected these species because they are typical representatives of nonwoody and woody vegetation that occurs naturally at the tree line where vegetation is presumably shaped by temperature limitations. After an initial conditioning period, we traced the fate of assimilates by pulse‐labelling the microcosms with ^14^CO_2_.

### 2.1 Plant material and microcosm preparation

The preparation of plant material and microcosms is detailed in Supporting Information. All seeds material originated from locations in the Swiss Alps near the treeline (2,000–2,200 m a.s.l.). Plants were transferred to cylindrical microcosms (10 cm diameter × 15 cm length) filled with soil collected at a treeline site where both species co‐occur. Microcosms were kept for 7 months (*L. alpina*) and 16 months (*P. mugo*) in a glasshouse at day and night temperatures of 12–14 and 8–10°C, respectively, with a photoperiod of 15 hr. Microcosms were watered regularly and supplied with mineral fertilizer.

### 2.2 Experimental design

The temperature treatment we applied consisted of average target temperatures of 4 and 9°C. This manipulation was applied to air and soil separately, creating four distinct temperature combinations. When exposed to this treatment, *L. alpina* and *P. mugo* plants were 8 months and 4 years old, respectively. By then, the roots of both species filled the whole soil compartment. *L. alpina* had multiple, heavily branched shoots with lengths of up to 10 cm. The apical shoot of *P. mugo* reached 10–12 cm in height.

The air temperature treatment and the isotope label were applied with the help of a large acrylic chamber that contained up to eight microcosms (Supporting Information). We had only a single such chamber, and therefore applied the different temperature manipulations in sequential runs. The specific air temperature levels were assigned randomly to consecutive pairs of runs. In total, there were four runs with *L. alpina* (2012, April 30th–July 7th) and eight runs with *P. mugo* (2013, February 4th–June 1st), that is, there were two and four replicates for each air temperature. The soil temperature treatment was applied to groups of microcosms within the chamber. Chamber construction and temperature control are described in Supporting Information.

### 2.3 Temperature treatment and ^14^CO_2_ pulse‐labelling

**Figure 1:**
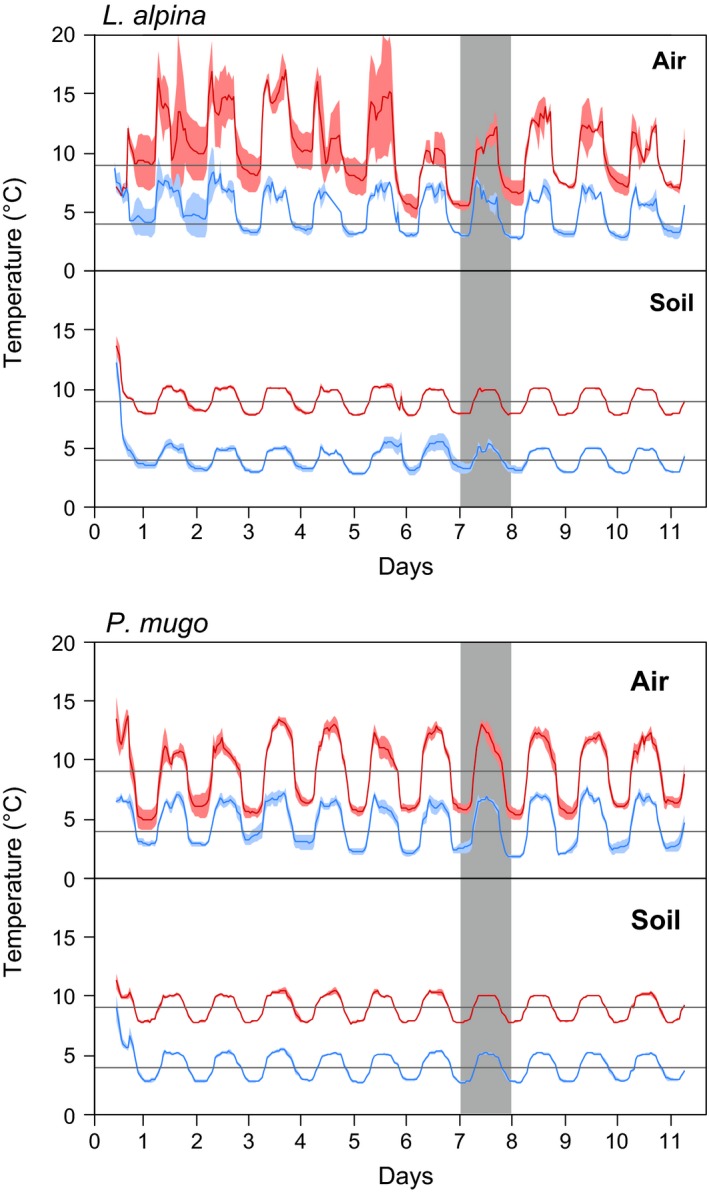
Air and soil temperature over the 11 days of experimental manipulation. Data show averages across blocks, with shaded areas indicating standard errors calculated using blocks as replicate (air temperature: *n* = 2 in *L. alpina* and *n* = 4 in *P. mugo*: soil temperature: *n* = 8 in *L. alpina* and *n* = 16 in *P. mugo)*. The gray area indicates the day of ^14^C labelling. Horizontal lines indicate target average temperatures (4 and 9°C)

The sides of the microcosms were insulated with 1 cm thick foam to restrict heat fluxes to the top and bottom surface and to prevent lateral gradients in soil temperature. Microcosms were well water‐supplied (60% water holding capacity (WHC), adjusted before the microcosms were placed in the chamber). The chamber was left slightly open to enable air exchange while still allowing for temperature regulation. The diurnal temperature amplitudes achieved were 6°C in air and 2°C in soils (Figure 1) and reflect typical growing‐season conditions found in the Swiss Alps at 2,200 m a.s.l. (Hagedorn et al., 2010). We regularly added water to compensate for water losses and re‐adjusted levels to 60% WHC after 7 days. Then, the chamber was sealed, and ^14^CO_2_ released by acidification of a Na_2_^14^CO_3_ solution in a glass bulb through which chamber air was circulated. The chamber was kept closed for 24 hr during which CO_2_ concentrations were kept between 300 and 500 ppm (LI‐6200, Licor, Lincoln, NE) by releasing CO_2_ from unlabeled Na_2_CO_3_ when required. This procedure maximized ^14^CO_2_ uptake. Then, the chamber was opened, vented, and the temperature treatment continued for another 4 days before the microcosms were harvested destructively.

To label *L. alpina* microcosms, we released 57.5 kBq ^14^C per microcosm (but note that the amount of label was controlled at the chamber and not the microcosm level; Supporting Information for a Discussion). Because some of the measured fractions had very low labelling, we later used higher amounts when labelling *P. mugo* microcosms; specifically, we released 100 kBq ^14^C per microcosm, or 650 kBq when the chamber included microcosms for the autoradiographic analysis of soil sections (see below).

### 2.4 Soil respiration

We collected soil respiration (CO_2_ and ^14^CO_2_) in the postlabelling phase of the experiment. In *L. alpina* microcosms, soil respiration was collected from hydrophobic gas‐permeable tubes (Accurel PP V8/2HF, Membrana GmbH, Wuppertal, Germany) installed horizontally at 5 and 10 cm depth, through which air (2 ml/min) was pumped. The collected CO_2_ was trapped in 0.5 M NaOH before the now CO_2_‐free air was circulated back to the tubing. We also trapped soil surface CO_2_ efflux but did not use these data because of technical difficulties. For the *P. mugo* microcosms that were labelled later we simplified the setup to a static microchamber (2.7 cm diameter × 6 cm length test tube inserted 3 cm into the ground) which contained a vial with 2 ml 1M NaOH.

NaOH solutions were replaced every 24 hr and the trapped CO_2_ quantified by acid titration after precipitation of carbonate with BaCl_2_, using phenolphthalein as indicator (Alef & Nannipieri, 1995). Trapped ^14^CO_2_ was determined by liquid scintillation counting of a 1 ml aliquot (TriCarb 2900, Packard BioScience, Meriden, CT; 4 ml Ultima Gold cocktail, Perkin Elmer, Waltham, MA).

### 2.5 Destructive harvest

Plant shoots were clipped at soil level. The microcosms used for autoradiographic imaging of belowground ^14^C distribution were immediately frozen and processed as described below. Soil and roots were collected from the remaining microcosms separately from 0–5, 5–10 and 5–15 cm depth sections. Roots were washed, the root‐free soil sieved (2 mm), and an aliquot stored at 4°C for microbial biomass determination. A separate root‐free soil aliquot was dried at 105°C for bulk ^14^C analysis. All plant material was dried at 75°C and weighed.

### 2.6 Analysis of plant and soil material

Dry plant and soil material was ground and ^14^C in samples quantified by liquid scintillation counting (LSC) of ^14^CO_2_ produced by dry combustion (Packard 307 sample oxidizer; 6 ml Carbosorb E mixed with 12 ml Permafluor E, Perkin Elmer).

Soil microbial C was determined by chloroform fumigation‐extraction (Vance, Brookes, & Jenkinson, 1987), with some modifications (Supporting Information). For *L. alpina*, ^14^C in microbial extracts was below the detection limit, most likely because of the lower amount of ^14^C applied during pulse‐labelling.

### 2.7 Autoradiography of soil sections

The frozen and structurally still intact belowground parts of the microcosms were freeze‐dried, embedded in epoxy resin, and a vertical soil section prepared that was oriented vertically through the center of the microcosm (Stiehl‐Braun et al., 2011; Supporting Information for details). This section was used to expose imaging plates that were then scanned at 200 μm resolution. We then recorded the depth distribution of the activity, excluding areas containing the highly‐labelled main root of *P. mugo* (Supporting Information Figure S5).

### 2.8 Statistical analyses

Given that response patterns differed among species and that these differences could not unequivocally be attributed to species identity because the species were labelled at different ontogenetic stages and times of the season, we analyzed these data sets separately. All data were analyzed by fitting linear models that reflected the hierarchical nature of the experimental design (aov function with error terms, http://www.r-project.org). The terms fitted were block, soil and air temperature (coded as two‐level factors), and the interaction of soil and air temperature. Block refers to replicates in time, that is, consecutive pairs of runs at low and high air temperature. The error terms fitted were run (the replicate for the air temperature treatment) and run × microcosm pair (the soil temperature treatment was randomly assigned to sets of two microcosms within the chamber). For the analysis of data available at the soil layer, the model was extended with interactions with soil depth, and the corresponding error terms (run × layer as error term for air temperature × depth, and run × microcosm pair × layer as error term for soil temperature × depth).

All dependent variables that quantified amounts of material (e.g., biomass, ^14^C in specific fractions) were log‐transformed prior to analysis (Supporting Information for Rationale and Implications). Nonsignificant interactions of air and soil temperature thus indicate that relative effects of soil temperature, that is, the percent change from 4 to 9°C, are independent of air temperature. Dependent variables that were ratios (e.g., the fraction of plant ^14^C that was allocated to roots) were analyzed untransformed because the calculation of the ratio already standardized for the total amount of label. Our experimental design did not allow for tests of air temperature effects on absolute amounts of ^14^C. The reason is that microcosms exposed to low and high air temperatures were labelled separately. The amount of ^14^C released in each run therefore largely determined total uptake so that differences in assimilation rates between 4 and 9°C air temperature could not manifest (Discussion in Supporting Information). However, the analysis of the proportional distribution of ^14^C among ecosystem compartments is unaffected by this caveat because it does not depend on label amounts. This limitation also does not apply to tests of soil temperature effects because the microcosms exposed to low and high soil temperature were exposed to the same atmospheric ^14^C concentrations, during the same labelling event.

## 3 RESULTS

We first analyzed effects of the temperature treatments in an overall model with both species. Despite some commonalities, specific response patterns differed among species to an extent that made interpretation and presentation difficult. Also, the experiment was not randomized at the level of species (*L. alpina* was labelled first, followed by *P. mugo*). We therefore present results from separate analyses. The main statistical results are summarized in Table 1.

**Table 1:** Statistical tests for effects of air temperature (Air), soil temperature (Soil), and their interaction (Air × Soil) in microcosms with either *Leucanthemopsis alpina* or *Pinus mugo*

Fraction	*L. alpina*						*P. mugo*					
	Air		Soil		Air × Soil		Air		Soil		Air × Soil	
	*df*, ddf	*F*‐value	*df*, ddf	*F*‐value	*df*, ddf	*F*‐value	*df*, ddf	*F*‐value	*df*, ddf	*F*‐value	*df*, ddf	*F*‐value
Plant biomass												
Total	1, 1	6.84 n.s.	1, 10	7.67 *	1, 10	1.04 n.s.	1, 3	0.07 n.s.	1, 14	0.82 n.s.	1, 14	0.87 n.s.
Shoots	1, 1	4.13 n.s.	1, 10	3.44 (*)	1, 10	0.22 n.s.	1, 3	0.00 n.s.	1, 14	0.17 n.s.	1, 14	1.49 n.s.
Roots	1, 1	0.20 n.s.	1, 10	6.60 *	1, 10	1.14 n.s.	1, 3	0.64 n.s.	1, 14	2.37 n.s.	1, 14	0.08 n.s.
Plant ^14^C												
Total	1, 1	2.15 n.s.	1, 10	4.62 (*)	1, 10	0.31 n.s.	1, 3	2.02 n.s.	1, 14	0.77 n.s.	1, 14	0.39 n.s.
Shoots	1, 1	1.66 n.s.	1, 10	3.22 n.s.	1, 10	0.32 n.s.	1, 3	7.88 (*)	1, 14	1.67 n.s.	1, 14	5.62 *
Roots	1, 1	0.05 n.s.	1, 10	15.05 **	1, 10	0.42 n.s.	1, 3	43.6 **	1, 14	0.00 n.s.	1, 14	2.10 n.s.
Root fraction	1, 1	0.03 n.s.	1, 10	4.62 (*)	1, 10	0.31 n.s.	1, 3	61.3 **	1, 14	2.86 n.s.	1, 14	20.2 ***
Soil ^14^C												
Total (excl. roots)	1, 1	8.62 n.s.	1, 10	8.64 *	1, 10	0.06 n.s.	1, 3	31.3 *	1, 14	0.18 n.s.	1, 14	1.35 n.s.
Soil microbial biomass		–	–		–		1, 1	0.38 n.s.	1, 8	0.00 n.s.	1, 8	0.22 n.s.
Soil depth dependency of temperature effects												
Root biomass	1, 2	5.10 n.s.	1, 10	5.04 *	1, 10	0.05 n.s.	1, 6	0.00 n.s.	1, 14	5.27 *	1, 14	6.89 *
Root ^14^ C	1, 2	0.21 n.s.	1, 10	88.42 ***	1, 10	0.10 n.s.	1, 6	1.87 n.s.	1, 14	0.05 n.s.	1, 14	3.67 (*)
Soil ^14^C	1, 2	2.17 n.s.	1, 10	6.43 *	1, 10	0.11 n.s.	1, 6	0.07 n.s.	1, 14	0.02 n.s.	1, 14	12.58 **

Note

Results are shown for the analysis of plant biomass (total = shoots + roots, shoots, and roots), the amount of ^14^C recovered in these plant fractions plus the root fraction of ^14^C (^14^C in roots relative to total plant ^14^C), and the amount of ^14^C recovered in soils (total, and soil microbial biomass for *P. mugo)*. For roots and soil ^14^C, tests for the temperature‐dependency of their depth‐distribution are provided (soil layers: 0–5 cm, 5–10 cm, and 10–15 cm). A significant soil temperature × depth interaction indicates a shift in depth distribution with soil temperature, for example, downwards with increasing temperature. All data were log‐transformed. *F*‐values are given with nominator and denominator degrees of freedom. ***p < 0.001, **p < 0.01, *p < 0.05, (*) *p* < 0.1, n.s. *p* > 0.1). Methods and Results for details.

### 3.1 *L. alpina* microcosms

#### 3.1.1 Plant biomass

**Figure 2:**
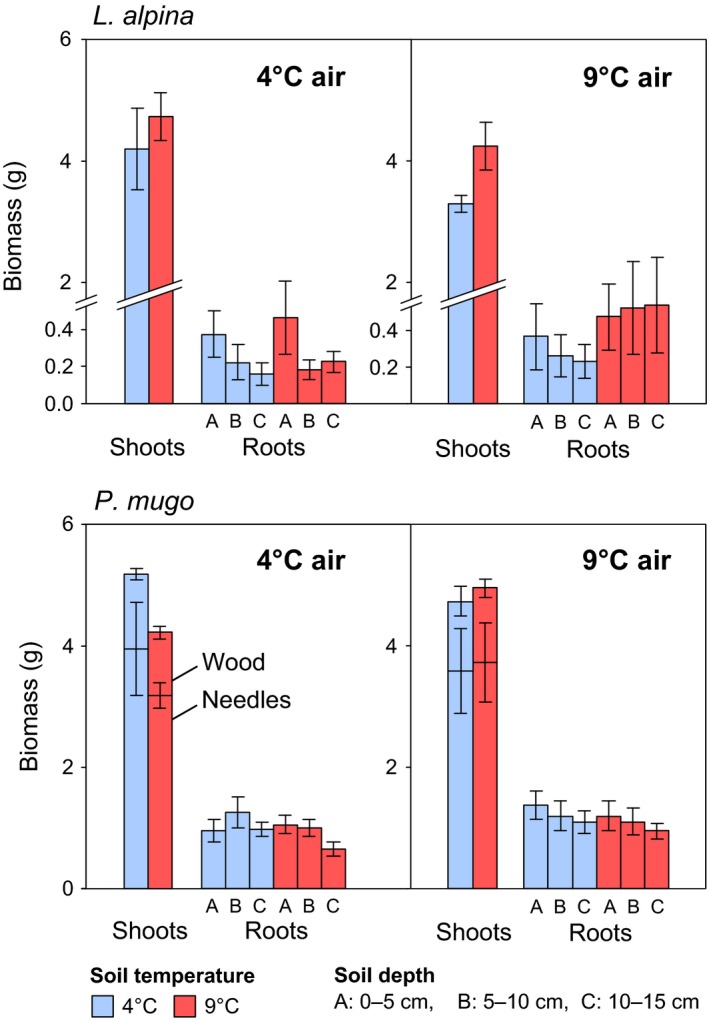
Effects of air and soil temperature on shoot and root biomass of 7‐month old *L. alpina* and 4‐year‐old *P. mugo* saplings. Error bars are standard errors (*n* = 8 for *L. alpina*,* n* = 12 for *P. mugo*)

Air temperature did not affect total plant biomass, that is, the sum of shoot and root mass. However, plant biomass was 20% lower at 4°C soil temperature than at 9°C (*p *<* *0.05, Figure 2). A similar effect was found for root mass (*p *<* *0.05).

#### 3.1.2 Distribution of ^14^C among plant and soil fractions

Total ^14^C recovered in the microcosms (plant plus soil material) averaged 40% of the activity released. Independent of air and soil temperature, 94% of the microcosm ^14^C was in plant biomass (6% in soil). Root ^14^C did not depend on air temperature but increased with soil temperature (*p *<* *0.01, Table 2). A similar trend (*p *<* *0.1) was found for the fraction of plant ^14^C in roots.

**Table 2:** Distribution of ^14^C recovered in microcosms at final harvest. Data show percentages of totals per labelling run (means ± *SE*)

Fraction	Temperature treatments				Treatment averages					
	4°C Air		9°C Air		Air			Soil		
	4°C Soil	9°C Soil	4°C Soil	9°C Soil	4°C	9°C	Δ%	4°C	9°C	Δ%
*L. alpina*										
Shoots	40.8 ± 7.8	47.9 ± 3.5	34.5 ± 4.4	53.0 ± 8.7	44.3 ± 0.1	43.8 ± 1.6	−1	37.6 ± 4.3	50.4 ± 4.5	+34
Roots	1.63 ± 0.32	4.24 ± 0.65	2.05 ± 0.81	3.69 ± 1.38	2.93 ± 0.05	2.87 ± 1.61	−2	1.84 ± 0.41	3.97 ± 0.71	+115
0–5 cm	1.11 ± 0.14	1.91 ± 0.33	1.43 ± 0.59	1.06 ± 0.32	1.51 ± 0.21	1.24 ± 0.57	−18	1.27 ± 0.29	1.48 ± 0.27	+17
5–10 cm	0.30 ± 0.10	0.98 ± 0.21	0.49 ± 0.22	1.48 ± 0.65	0.64 ± 0.11	0.98 ± 0.65	+54	0.39 ± 0.12	1.23 ± 0.33	+212
10–15 cm	0.22 ± 0.08	1.35 ± 0.40	0.13 ± 0.04	1.16 ± 0.48	0.79 ± 0.15	0.65 ± 0.39	−18	0.18 ± 0.04	1.26 ± 0.29	+612
Soil	1.86 ± 0.31	3.66 ± 0.70	2.45 ± 0.48	4.25 ± 0.69	2.76 ± 0.09	3.35 ± 0.05	+21	2.15 ± 0.29	3.96 ± 0.47	+84
0–5 cm	0.95 ± 0.19	1.35 ± 0.16	0.98 ± 0.23	1.15 ± 0.31	1.15 ± 0.03	1.07 ± 0.05	−7	0.96 ± 0.14	1.25 ± 0.16	+30
5–10 cm	0.39 ± 0.11	1.06 ± 0.33	0.72 ± 0.15	1.37 ± 0.23	0.72 ± 0.02	1.05 ± 0.14	+45	0.55 ± 0.11	1.22 ± 0.20	+119
10–15 cm	0.52 ± 0.13	1.26 ± 0.24	0.75 ± 0.20	1.72 ± 0.22	0.89 ± 0.13	1.24 ± 0.03	+39	0.64 ± 0.12	1.49 ± 0.18	+134
*P. mugo*										
Shoots	45.7 ± 8.0	33.2 ± 5.2	33.5 ± 5.6	36.4 ± 6.2	44.4 ± 8.8	38.9 ± 7.7	−12	39.6 ± 5.0	34.8 ± 3.9	−12
Roots	19.26 ± 4.79	22.23 ± 3.71	31.00 ± 7.93	23.11 ± 3.92	23.54 ± 5.38	31.11 ± 7.99	+32	25.13 ± 4.76	22.67 ± 2.58	−10
0–5 cm	5.81 ± 0.95	9.94 ± 1.90	15.99 ± 5.05	10.67 ± 2.17	8.78 ± 1.66	15.81 ± 4.99	+80	10.90 ± 2.89	10.30 ± 1.38	−6
5–10 cm	9.17 ± 3.47	7.49 ± 1.55	9.42 ± 2.08	7.00 ± 0.87	9.92 ± 3.15	9.08 ± 1.85	−8	9.29 ± 1.93	7.25 ± 0.85	−22
10–15 cm	4.28 ± 0.70	4.80 ± 0.48	5.59 ± 1.42	5.44 ± 1.21	4.84 ± 0.64	6.22 ± 1.25	+28	4.93 ± 0.78	5.12 ± 0.63	+4
Soil	6.86 ± 0.91	6.10 ± 0.97	4.59 ± 0.67	4.74 ± 0.42	7.03 ± 1.29	4.99 ± 0.60	−29	5.73 ± 0.64	5.42 ± 0.55	−5
0–5 cm	3.04 ± 0.90	3.77 ± 1.15	2.04 ± 0.36	1.92 ± 0.40	3.90 ± 1.43	2.01 ± 0.31	−48	2.54 ± 0.49	2.85 ± 0.64	+12
5–10 cm	2.32 ± 0.40	1.15 ± 0.13	1.80 ± 0.46	1.65 ± 0.39	1.83 ± 0.24	2.02 ± 0.53	+10	2.06 ± 0.30	1.40 ± 0.21	−32
10–15 cm	1.51 ± 0.15	1.17 ± 0.26	0.76 ± 0.13	1.17 ± 0.05	1.30 ± 0.19	0.96 ± 0.11	−26	1.13 ± 0.15	1.17 ± 0.13	+3
Soil microbes	4.30 ± 1.51	3.86 ± 1.36	2.98 ± 1.05	2.66 ± 0.84	4.93 ± 1.55	2.93 ± 1.32	−41	3.64 ± 0.90	3.26 ± 0.78	−10

Note

For roots and soil organic matter, data are given as totals and separately for the three depth layers (0–5, 5–10, and 10–15 cm). A labelling run includes a single air temperature but two replicates for each soil temperature. The sum of shoots, roots, and soil is 100 (%) for each air temperature. The standard errors provided are based on the statistical replicates, which are pairs of microcosms with equal soil temperature except for air temperature averages where replicates are labelling runs. Methods and Supporting Information for details.

#### 3.1.3 Vertical distribution of ^14^C in roots and soil

Root biomass decreased with soil depth (*F*_1,10_ = 11.3, *p *<* *0.01, Figure 2). This decrease was independent of air temperature but stronger in cold soils (*p *<* *0.05 for depth × soil temperature). Root ^14^C also decreased with depth (Table 2, *F*_1,10_ = 148.4, *p* < 0.001), independent of air temperature. However, the root ^14^C decrease with depth was only pronounced at 4°C soil temperature with only a small gradient at 9°C (*p *<* *0.001, Table 1, Table 2).

**Figure 3:**
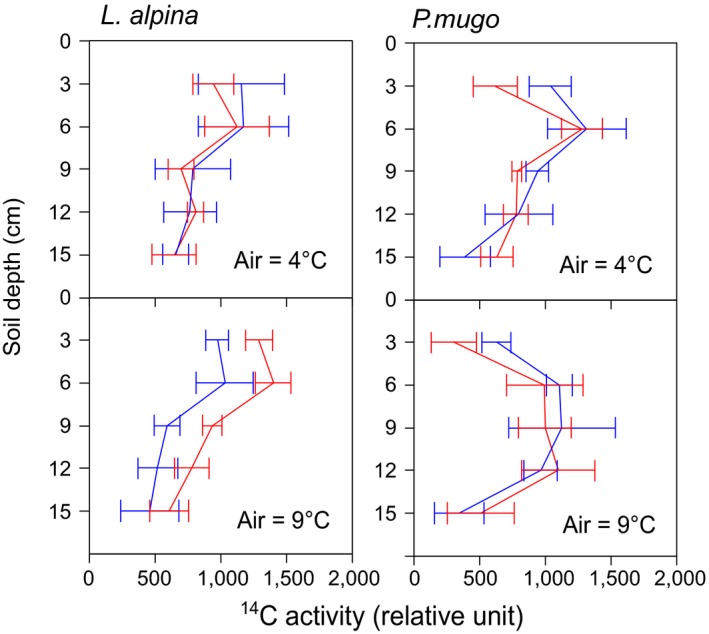
^14^C distribution over the soil profile, determined by autoradiography (Supporting Information Figure S5 for image examples); data were averaged to 3 cm depth layers. Error bars are standard errors (*n* = 6 for *L. alpina*,* n* = 8 for *P. mugo*)

Soil ^14^C, that is, net rhizodeposition, approximately followed the distribution of root ^14^C (Table 2) and showed similar temperature effects (air temperature: n.s.; soil temperature × depth: *p *<* *0.05). No soil temperature × depth interactions were found for ^14^C recorded in autoradiographies (Figure 3).

#### 3.1.4 Soil respiration

**Figure 4:**
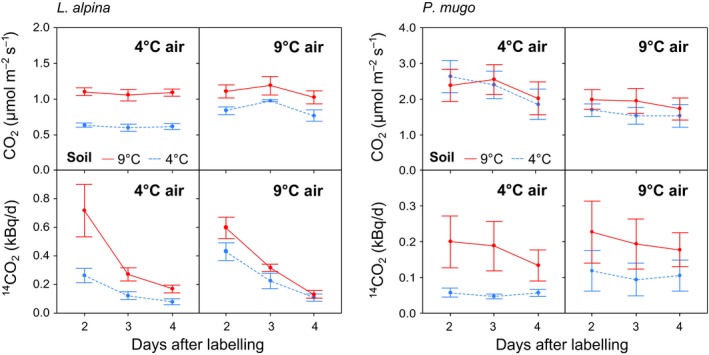
Air and soil temperature effects on soil CO_2_ and ^14^ CO_2_ efflux in microcosms planted with *L. alpina* and *P. mugo*. Soil respiration was trapped over 24 hr intervals. Data for the first 24 hr after pulse‐labelling (day 1) are not available because respired CO_2_ could not be collected without contamination during labelling. Error bars are standard errors (*n* = 8 for *L. alpina*,* n* = 16 for *P. mugo*)

Soil CO_2_ efflux increased with soil temperature (Figure 4, *p* < 0.001), and this effect tended to be larger at low than at high air temperature (*p *<* *0.05, for soil × air temperature). Soil ^14^CO_2_ efflux also increased with soil temperature (*p *<* *0.01), independent of air temperature. Soil ^14^CO_2_ efflux decreased rapidly over time (Figure 4). We quantified temporal decay rates of ^14^CO_2_ evolution by fitting a first‐order exponential decay curve; decay rate constants did not vary with air or soil temperature (mean of *k* = 0.76 d^−1^).

### 3.2 *P. mugo* microcosms

#### 3.2.1 Plant biomass

Neither air temperature nor soil temperature affected total plant biomass, shoot biomass, or root biomass (Figure 2).

#### 3.2.2 Distribution of ^14^C among plant and soil fractions

Total ^14^C recovery in the microcosms (plant plus soil material) averaged 46% of the activity originally released. This fraction was independent of air and soil temperature. Independent of air and soil temperature, 91% of microcosm ^14^C were recovered in plant biomass (9% in soil). A number of statistically significant effects of temperature occurred for plant ^14^C fractions (Table 1). These were largely driven by a root ^14^C increase with air temperature that only occurred when soils were at 4°C and manifested in significant main effects of air temperature and interactions between air and soil temperature on root ^14^C and the root fraction of plant ^14^C (Table 2). The fraction of microcosm ^14^C recovered in soil decreased with air temperature (*p* < 0.05) but was not affected by soil temperature. Microbial biomass accounted for 62% of total soil ^14^C (Table 2) with no effects of air and soil temperature.

#### 3.2.3 Vertical distribution of ^14^C in roots and soil

Root biomass slightly decreased with soil depth (*p *<* *0.05, Figure 2), independent of air and soil temperature. Root ^14^C decreased steeply with soil depth in all air and soil temperature combinations (*p* < 0.001), but this effect was less regular when both air and soil were at 4°C and ^14^C amounts were highest in the middle soil layer. Soil ^14^C approximately followed the distribution of root ^14^C (Table 2); similar to root ^14^C, soil ^14^C decreased least when both air and soil were cold; this manifested in a significant depth × air temperature × soil temperature interaction (*p* < 0.01).

The autoradiographies of belowground sections (Figure 3) revealed that ^14^C depth distribution depended on both air (*F*_4,46_ = 2.81, *p *<* *0.05 for depth × air temperature) and soil temperatures (*F*_4,46_ = 4.67, *p *<* *0.01 for depth × soil temperature). Mean ^14^C allocation depth increased with soil (but not with air) temperature (*F*_1,7_ = 13.4, *p *<* *0.01) from 5.9 to 6.9 cm.

#### 3.2.4 Soil respiration

Soil CO_2_ efflux was slightly higher in warm soils (Figure 4), but independent of air temperature. Soil ^14^CO_2_ efflux increased with soil temperature (*p *<* *0.01), but remained unaffected by air temperature. The rate of decrease in soil ^14^CO_2_ efflux over time (Figure 4) was independent of air and soil temperature, although there was a statically nonsignificant trend toward a higher decay rate constants in warmer soils (*k* = 0.009 ± 0.061 in cold and 0.091 ± 0.056 in warm soils).

## 4 DISCUSSION

Do air or soil temperature control C allocation in cold environments? We independently manipulated air and soil temperature in experimental microcosms of a nonwoody forb (*Leucanthemopsis alpina*) and of a tree (*Pinus mugo*) that both occur naturally at the alpine treeline. While some of the responses that we observed where species‐specific, the general pattern that emerged was that C allocation was more strongly affected by belowground than by aboveground temperature. Our findings therefore suggest that the ultimate mechanisms that control C allocation within the plant, and also the subsequent turnover of rhizodeposits in the soil, are primarily located below ground. Our experiment further demonstrates that these processes are sensitive to temperature changes in the range we studied (approx. 2–10°C). At the alpine treeline, such temperatures are frequently reached in the shoulder season but also around night‐time during peak growing season or during cold spells, indicating that these effects are ecologically relevant.

Low soil temperatures restricted C cycling, in particular belowground. One of the clearest manifestations of this limitation was that the release of ^14^CO_2_ strongly increased with soil temperature, in microcosms with both species. Given that plant aboveground and belowground physiological processes are strongly linked, the ultimate physiological drivers of this temperature dependency cannot unambiguously be identified. However, there is compelling evidence that root metabolism is temperature‐dependent (Ericsson, Rytter, & Vapaavuori, 1996; Iivonen, Rikala, Ryyppo, & Vapaavuori, 1999; Pregitzer, King, Burton, & Brown, 2000). In particular, root growth virtually ceases when soil temperatures drop below approximately 6°C (Alvarez‐Uria & Körner, 2007; Schenker et al., 2014; Vapaavuori, Rikala, & Ryyppo, 1992). This mechanism easily explains the temperature effects that we observed. Plant growth, and therefore also root growth, obviously also depend on photosynthesis. However, leaf temperatures are remarkably decoupled from air temperatures (Helliker & Richter, 2008). In hot environments, transpiration substantially reduces leaf temperatures. Conversely, the heating of leaves by absorbed solar radiation is more important in cold environments (Körner, 2003). We did not measure leaf temperatures in our study. Also, our experimental setup did not allow quantifying air temperature effects on the net assimilation of labelled CO_2_ because net uptake was largely determined by the amount of label released (Methods for details). Nevertheless, air temperatures were well above the freezing point and radiation from overhead lamps was strong, so that we consider it unlikely that air temperature limited CO_2_ assimilation. However, net assimilation may be reduced indirectly when soil temperatures are low because a low root activity will reduce belowground assimilate consumption (Domisch, Finer, & Lehto, 2001; Hoch & Körner, 2009; Hoch et al., 2002; Kontunen‐Soppela, Lankila, Lähdesmäki, & Laine, 2002).

Another mechanism by which plant growth may be limited at low soil temperature is reduced nutrient supply from organic matter mineralization (Dieleman et al., 2012; Dormann & Woodin, 2002; Melillo, Steudler, Aber, & Newkirk, 2002). Soil microbial activity generally drops at lower temperatures, but how closely the supply of available N to plants tracks the effects of temperature on decomposition is less clear, because net N mineralization depends on microbial turnover through both mineralization and immobilization processes. In our study, exposure of microcosms to the different temperature treatments was short and we therefore think that it is unlikely that soil warming caused a substantial increase in N availability. Furthermore, a general pattern found in plants is that they respond to a shortage of mineral nutrients by increasing root growth relatively to shoot growth. In our study, however, we observed a reduced ^14^C allocation belowground which suggests that belowground plant activity was limited by factors other than nutrients when soils were cold. This reasoning is compatible with the notion of a temperature‐limitation on tissue growth as was postulated by Körner (1998).

In a soil warming experiment at the alpine treeline, we recently have shown that the temperature sensitivity of rhizosphere respiration is higher than that of total soil respiration between 5 and 10°C soil temperature, but smaller between 10 and 15°C (Ferrari, Hagedorn, & Niklaus, 2016). The pattern we have found between 10 and 15°C is in line with many other studies that also have shown a higher temperature dependency of total soil respiration compared to root and rhizosphere respiration (Hagedorn et al., 2010; Hartley, Heinemeyer, Evans, & Ineson, 2007; Streit et al., 2014; Vogel, Bronson, Gower, & Schuur, 2014; Wang et al., 2014). These seemingly conflicting patterns can be reconciled by assuming that soil microbial activity is generally more temperature sensitive than root activity, except in the temperature range around 4–8°C where root growth ceases relatively abruptly and the pattern therefore is reversed.

In natural ecosystems, soil temperature follows a depth gradient. An interesting consequence of a strong temperature limitations of assimilate investment into roots in cold soil is that soil temperature changes will lead to a change in the depth in which organic matter is deposited. The specific patterns are complicated to predict because soil temperature gradients often reverse during diurnal and seasonal cycles. Soil temperature at depth is relatively well buffered and integrates heat budgets (convective, conductive, and radiative fluxes) over longer time scales. Closer to the soil surface, temperatures are higher at daytime and early in the growing season, whereas the opposite occurs at night and at the end of the season. In our experiment, soil temperatures were relative homogenous over the entire soil profile, varying only over the upper 2–3 cm of soil when air and soil temperatures differed. A direct effect of the air treatment on soil temperatures can therefore be excluded. The root and soil ^14^C we found in combusted samples and in autoradiographies indicate that C is deposited at larger depth when soils are warmer. This is likely driven by stronger C sink activity in warmer soils, while reduced C sink‐strength in cold soil reduced the downwards transport of recent assimilates.

Many responses to the temperature treatments differed between *L. alpina* and *P. mugo*. While woody and nonwoody vegetation clearly differ in functional traits, the two species also were labelled at different times in the growing season and were in different ontogenetic stages. It therefore is not possible to attribute the different effects we found to growth form only (forb vs. tree). A generalization would require a setup with a larger number of species, and ideally multiple labeling events throughout the season and with plants of different age. Nevertheless, our findings suggest that species‐specific responses may contribute to the conflicting observations of temperatures sensitivities of autotrophic and heterotrophic soil respiration (Janssens & Pilegaard, 2003; Schindlbacher, Zechmeister‐Boltenstern, & Jandl, 2009).

Our study addressed short‐term effects of air and soil temperature of C allocation and the processing of rhizodeposits. Extrapolating these to growth and longer time scales is difficult. Some of the applied label will have been in nonstructural fractions used as carbohydrate stores. Whether these would eventually have been allocated to growth in the same organ is unclear. Over longer time scales, effects also could be modified, for example by acclimation. At the population and community level, responses might change further because other genotypes or species are favored. At the ecosystem level, feedback mechanisms including carbon and nutrient dynamics will modify the initial effects we addressed. For example, many studies have found that warming effects on soil respiration decreased with time (Carey et al., 2016; Luo, Wan, Hui, & Wallace, 2001; Romero‐Olivares, Allison, & Treseder, 2017). The mechanisms that have been put forward as explanation include the acclimation of root metabolism (Atkin, Edwards, & Loveys, 2000; Burton, Melillo, & Frey, 2008), the exhaustion of labile soil organic matter pools that fuel microbial respiration (Caprez, Niklaus, & Körner, 2012; Eliasson et al., 2005), and a thermal adaption of soil microbial communities (Bradford et al., 2008; Heinemeyer, Ineson, Ostle, & Fitter, 2006).

In summary, our study suggests that soil temperature is a more important controller of C allocation in cold ecosystems than air temperature. Most of the patterns that we found were compatible with the idea that root metabolism is strongly inhibited below a critical temperature between 4 and 9°C. Given the increasing frequency of extreme meteorological events, which are often associated with a decoupling of above‐ and belowground temperatures, the understanding of both short‐term and long‐term temperature responses appears important for predictions of ecosystem responses to warming. Our results thus emphasize that air and soil temperature variation must be considered separately when assessing ecosystem responses to global change, both in field warming experiments and in numeric models used to simulate plant and ecosystem performance in a future climate.

## AUTHOR CONTRIBUTIONS

AF set up the experiment and carried out all laboratory and data analyses, with assistance from PAN. AF wrote the manuscript, with contributions from FH and PAN. FH and PAN conceived the study and wrote the grant that funded the project.

## DATA ACCESSIBILITY

All data shown in this article (plant and microbial biomass, 14C distribution, soil and air temperatures) are deposited under Dryad https://doi.org/10.5061/dryad.mk1vd47.
