# Supplementary material for: Disentangling effects of air and soil temperature on C allocation in cold environments: A 14C pulse‐labelling study with two plant species
Source: Ecol Evol. 2018 Jul 13;8(16):7778–89. doi: 10.1002/ece3.4215 (PMC6144959; doi:10.1002/ece3.4215)
Supplement: Supplementary file 1 [file ECE3-8-7778-s001.pdf]

Supporting Information 1

1 Plant material and microcosm preparation

All seeds material originated from locations in the Swiss Alps near the treeline (2000-2200 m a.s.l.). Four-week old *L. alpina* seedlings were provided by Schutz-Filisur Alpin Gartencenter (Filisur, Switzerland). *P. mugo* was obtained from an experimental garden (WSL Birmensdorf, Switzerland) where saplings had been grown for two years. We transferred these plants to microcosms filled with sieved (7 mm mesh) soil from the organic horizon collected at a treeline site where both species co-occur (Stillberg, 46°46′20″N, 9°51′56″E, 2200 m a.s.l., see Table 1 in Hagedorn et al. 2010 for soil properties). The microcosms were made from 10 cm inner diameter × 15 cm length polyethylene tube sections (Geberit AG, Jona, Switzerland) that were closed at the bottom with a 2 mm nylon mesh (Fig. 1). Microcosms were kept for 7 months (*L. alpina*) and 16 months (*P. mugo*) in a glasshouse provided with a cooling system (Fig. 2). Day and night temperatures were 12–14 and 8–10°C, respectively. A photoperiod of 15 h was achieved with additional illumination that was turned on when natural ambient light dropped below 40 klx. Microcosms were watered three times per week. Once a week, soil moisture was adjusted to 60% of soil water holding capacity by weighing the microcosms. Plants were fertilized once a week with about 5–7 mg N, 2–3 mg P and 4–6 mg K per plant (liquid fertilizer NPK 8+8+8, Gesal, Allschwil, Switzerland).

2 Temperature control

Air temperature in the acrylic incubation chamber (120 cm length × 54 cm width × 52 cm height) was controlled by means of two fan-equipped radiators mounted at both ends inside the chamber (Fig. 3). Target air temperatures were achieved by periodically adjusting the flow of a 50% aqueous ethylene glycol solution that was circulated through the radiators (visible at the left and right in Fig. 3) from a 200 L cooling tank (Pro-Inox CV-G 200, Galactea, Cleppe, France, set to 1°C day and -2.5°C night-time temperature). To manipulate soil temperature, the bottom 2.8 cm of each microcosm was tightly fit into an aluminum block through which a coolant was circulated (Fig. 3). The block also contained a 100 W heating element. A custom-built electronic circuit and corresponding software were used to measure temperatures with probes inserted into the lower part of each microcosm; measurement of the soil surface of the microcosms indicated that the probes were 10.7 cm below the soil surface. A P-I-D-algorithm was used to control power delivery to the heating elements so that target temperatures were reached.

3 Rationale of labelling setup

An important consideration in any experiment is that the treatments that are to be compared are exposed to the same conditions except for the experimental manipulation. In a $^{14}CO_2$ labelling study, both the total CO_2 concentration and the specific ^{14}C activity of the CO_2 therefore need to be kept equal in the different treatments. Two popular possibilities to achieve this are:

1. One or several open flow-through chambers that harbor one or all replicates. The key point here is that air flowing through the chamber is not recycled back to the chamber. If single replicates are labelled separately, this requires a stable control of CO_2 and $^{14}CO_2$ concentrations to keep conditions comparable across replicates and treatments.

2. A common chamber in which $^{14}CO_2$ is released as a single pulse or gradually. Since all treatments are in the same chamber, unstable $^{14}CO_2$ concentrations will affect all treatments in the same way, at least if the chamber air is well mixed.

Option 2 is popular because it is easier to realize. Simple implementations consist of packing a plant, a pot, or a tree branch into a bag and injecting a defined amount of label into this bag. This approach has the advantage that $^{14}CO_2$ can be released as a pulse and the chamber kept close for a while to maximize uptake of the typically expensive label. However, under these conditions, it is critically important to harbor the treatments that are to be compared in the same chamber. Otherwise, the different treatments likely will experience different labelling conditions in terms of CO_2 and $^{14}CO_2$ concentrations. The reason is that assimilation of the label reduces CO_2 and $^{14}CO_2$ concentrations. CO_2 and $^{14}CO_2$ further are released to the chamber by plant and microbial respiration. Since assimilation and respiration rates will likely systematically differ among treatments, this would result in systematically different labelling conditions during the time the chamber is kept close.

A second reason why treatments need to be labelled in the same chamber is that, given sufficiently high assimilation rates, labelling extents tend to be determined by the amount of label released rather than by assimilation rates. In other words, differences in assimilation rates will not manifest fully in different label concentrations. Label uptake rates generally are positively correlated with label concentrations. Label assimilation thus reduces label concentrations and this in turn reduces uptake rates of the remaining label. An extreme case is reached when (virtually) all label is consumed during the time the chamber remains closed, independent of differences in net assimilation rates (example: in a cold soil treatment, 90% of the label is absorbed within 6 hours; with warmed soils, this happend in 3 hours, i.e. at twice the rate. However, the labelling chamber is kept closed for 12 hours so that the amount taken up in by both treatments is virtually identical).

In our study, we chose to place microcosms with low and high soil temperature in the same labelling chamber. All microcosms therefore experienced identical CO_2 and $^{14}CO_2$ concentrations, regardless of their soil temperature (the chamber was mixed with six fans). Of course, $^{14}CO_2$ concentrations decreased through time since CO_2 (and $^{14}CO_2$) was assimilated by the microcosms. Importantly, the microcosms of the different treatments competed for the $^{14}CO_2$ in the chamber atmosphere. If, for example, systems with warm soils assimilated CO_2 at twice the rate of systems with cold soils, then the amounts of label bound in the warm soil microcosms will have been twice as high as in the cold soil microcosms. Conversely, had one labelled the same warm and cold soil microcosms in separate chambers, both would have bound near equal amounts of label if one had left the chamber closed until most of the label had been assimilated.

Obviously, we were not able to place the two air temperature treatments in the same labelling chamber because mixing air would have equalized air temperatures. Instead, we applied the low and high air temperature treatment in subsequent labelling runs (see Table 1). As consequence, the amounts of label recovered in low and high air temperature microcosms does not reflect differences in assimilation rates at low and high air temperature (because, as explained above, the extent of labelling is largely determined by the amount of label released). However, the *relative* distribution of label *within the chamber* (i.e. among warm and cold soil microcosms, within microcosm among plant and soil, or within plant among roots and shoots) is unaffected by this caveat. We thus were, for example, able to test whether warm and cold soil microcosms differed in CO_2 uptake rates at a given air temperature, and whether these differences changed, relatively, with air temperature. We were, however, unable to test whether uptake rates changed with air temperature given a soil temperature.

For the statistical analyses, we *log*-transformed all ^{14}C activity data. All ^{14}C amounts were thus analysed on a proportional scale. We illustrate this point with a simple hypothetical example: we assume that warm soil systems assimilate 50% more CO_2 than cold soil systems. The distribution of label among cold and warm soil systems will thus be 40:60. We further assume that in both warm and cold soil systems plants allocate $\frac{1}{4}$ of the label to roots, and $\frac{3}{4}$ to shoots. The resulting overall label distribution will thus be as follows:

Soil	Plant uptake	Plant part	Amount	$\log_{10}$(Amount)	Difference (*log*-scale)
cold	40%	shoot	30%	1.477	Δ=0.477
		root	10%	1.000	
warm	60%	shoot	45%	1.653	Δ=0.477
		root	15%	1.176	

Note that on a *log*-scale differences between roots and shoot reflect root:shoot ratios, independent on the extent of labelling of cold and warm soil systems. Similarly, the results shown in main manuscript Table 1 reflect differences in relative label distribution that are independent of labelling extent. For example, a statistically significant interaction of air and soil temperature indicates that relative differences between soil temperature treatments depend on air temperature. This test is independent of the total amount of label applied to low and high air temperature microcosms that were labelled in different "labelling runs".

In summary, we optimized our design to compare C uptake rates and allocation between microcosms that differed in soil temperature. We are able to analyze effects of air temperature, and interactions of air and soil temperature with respect to the *relative* label distribution among soil temperature treatments, and within microcosms, but not with respect to C uptake rates.

4 Soil microbial biomass

Soil microbial C was determined by chloroform fumigation-extraction (Vance et al 1987), with some modifications. We replaced K_2SO_4 by Na_2SO_4 and reduced the concentrations of the extractant to avoided interferences from background radiation of ^{40}K and from high ionic strengths. In brief, 10 g soil were extracted with 30 mL 0.05 M Na_2SO_4 (45 min, 150 rpm), the suspension filtered (MN 615, Macherey-Nagel AG, Oensingen, Switzerland) and ^{14}C in the filtrate quantified by liquid scintillation counting. A second sample was processed similarly after fumigation with ethanol-free chloroform. Organic carbon concentrations in the extracts were measured with a TOC analyzer (Dimatoc 2000; Dimatec Analysentechnik GmbH, Germany). Microbial C and 14C were calculated assuming an extraction efficiency of k_{EC}=0.45 (Wu et al., 1990).

5 Soil sections

The frozen and structurally still intact belowground parts of the microcosms were freeze-dried and embedded in epoxy resin (Stiehl-Braun et al., 2011). First, we sealed the bottom of the microcosms with a thin layer of epoxy resin (Laromin C 260, BASF, Ludwigshafen, Germany, mixed at a volume ratio of 2:3 with Araldite DY 026 SP hardener, Astorit AG, Einsiedeln, Switzerland). Then, fresh resin was added from the top and air removed from soil pores by slowly evacuating the cores to $\approx$20 kPa in a desiccator and bringing pressure back to atmospheric levels (Stiehl-Braun et al., 2011). The resin was left curing at room temperature for three days and then fully hardened for 24 hours at 60°C. The resin cores was then extract from the microcosms and a 6 mm thick vertical section separated from the center of each resin core using a diamond saw (Discoplan TS, Struers GmbH, Birmensdordf, Switzerland). This section was divided into four rectangular pieces approx. 5 $\times$ 7 cm in size that were mounted on glass slides, milled flat with a diamond cup mill, and used to expose phosphor image plates (BAS III S, Fujifilm, Tokyo, Japan; 10 and 7 days exposure for *L. alpina* and *P. mugo* soils). The imaging plates were scanned (200 µm resolution, Fujix BAS 1000 scanner, Fujifilm, Tokyo, Japan) and the four images recomposed. Background exposure was subtracted from the data and the depth distribution of the recorded activity determined. For this analysis, areas containing the highly-labelled main root of *P. mugo* were excluded since their occurrence strongly depended on where the soil core had been cut.

6 Supplementary References

1. Hagedorn F, Martin M, Rixen C, Rusch S, Bebi P, Zurcher A, Siegwolf RTW, Wipf S, Escape C, Roy J, Hättenschwiler S (2010) Short-term responses of ecosystem carbon fluxes to experimental soil warming at the Swiss alpine treeline. *Biogeochemistry*, 97, 7–19.
2. Stiehl-Braun PA, Powlson DS, Poulton PR, Niklaus PA (2011) Effects of N fertilizers and liming on the micro-scale distribution of soil methane assimilation in the long-term Park Grass experiment at Rothamsted. *Soil Biology and Biochemistry*, 43, 1034–1041.
3. Vance E, Brookes P, Jenkinson D (1987) An extraction method for measuring soil microbial biomass C. *Soil Biology and Biochemistry*, 19, 703–707.
4. Wu J, Joergensen RG, Pommerening B, Chaussod R, Brookes PC (1990) Measurement of soil microbial biomass C by fumigation-extraction. An automated procedure. *Soil Biology and Biochemistry*, 22, 1167–1169.

7 Supplementary Tables

Species	Block	Run	Temperature	
			Air	Soil
L. alpina	A	Run 1	4°C	4°C & 9 °C
		Run 2	9°C	4°C & 9 °C
	B	Run 3	4°C	4°C & 9 °C
		Run 4	9°C	4°C & 9 °C
P. mugo	C	Run 5	4°C	4°C & 9 °C
		Run 6	9°C	4°C & 9 °C
	D	Run 7	9°C	4°C & 9 °C
		Run 8	4°C	4°C & 9 °C
	E	Run 9	9°C	4°C & 9 °C
		Run 10	4°C	4°C & 9 °C
	D	Run 11	4°C	4°C & 9 °C
		Run 12	9°C	4°C & 9 °C

Table 1: Experimental Design. *L. alpina* and *P. mugo* were labelled in separate, subsequent experiments. Air temperature was varied at the level of runs. Two pairs of runs with contrasting air temperature treatment formed a block. Soil temperature was varied at the level of microcosm pairs within each run. There were 4 pairs of microcosms (total of eight positions) within the chamber that was used for the individual runs (see Fig. 3).

8 Supplementary Figures

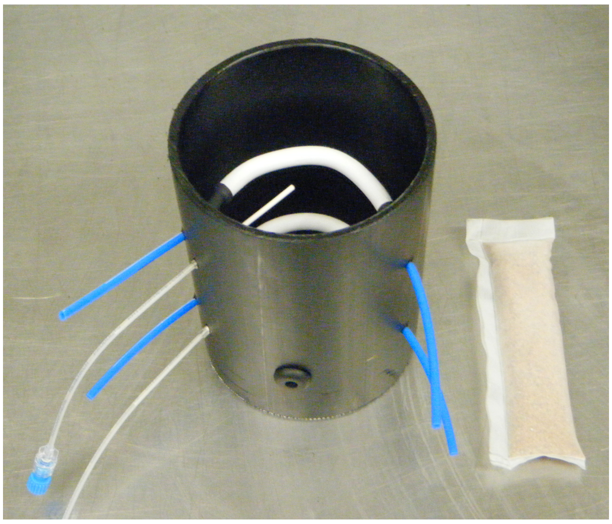

Figure 1: Microcosm tube with gas permeable tubes (thick white bent tubes) used for the collection of soil respiration at two depth in *L. alpina* micrososms. These tubes were later omitted when labelling *P. mugo* microcosms. In these systems, soil respiration was captured with a static chamber placed onto the soil surface. The hyphal ingrowth bag shown on the right was inserted vertically into the soil when the microcosms were filled with soil. The white pin-like probes are micro-suction cups that were used to collect soil solution. We were not able to trap detectable amounts of ^{14}C with these tubes and data are therefore not shown. Temperature probes were inserted through the black O-ring visible at the bottom of the tube wall.

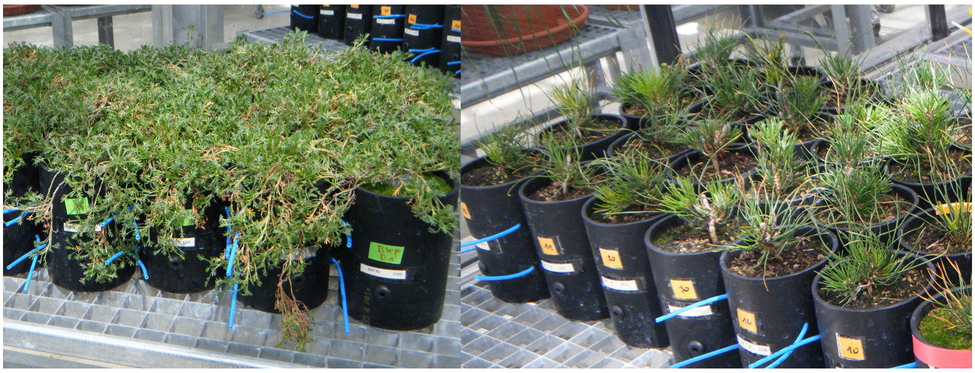

Figure 2: *L. alpina* (left and *P. mugo* (right) growing in microcosms.

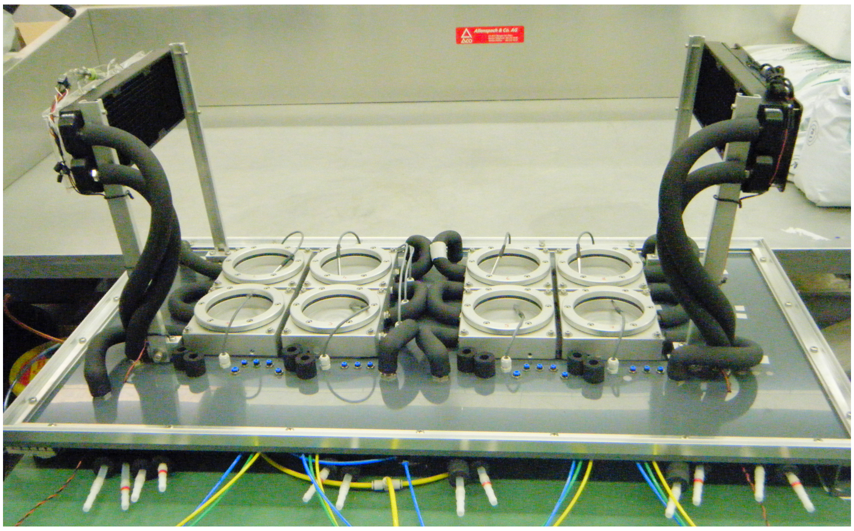

Figure 3: Labelling chamber (top removed) showing the soil temperature control plates into which the microcosms were inserted. Note the total of eight available positions. Two positions each were coupled with respect to the temperature treatment. We refer to these as “microcosm pairs” which form the replicates for the soil temperature treatment.

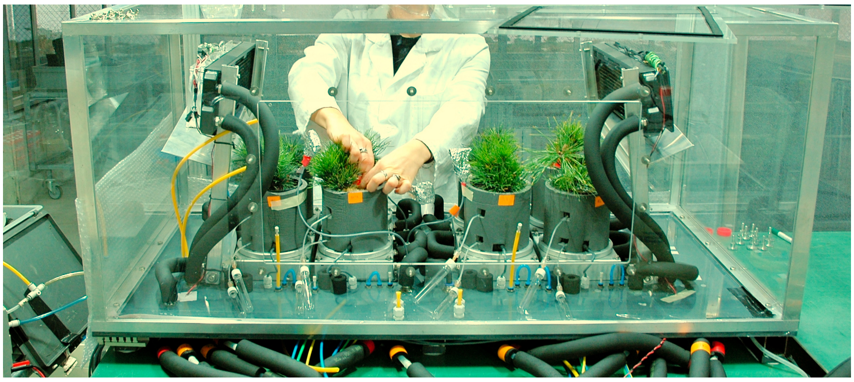

Figure 4: Preparation of a labelling run with *P. mugo* microcosms. Note that the microcosms are wrapped in a foam tube to prevent lateral temperature gradients. The soil temperature probe is already inserted.

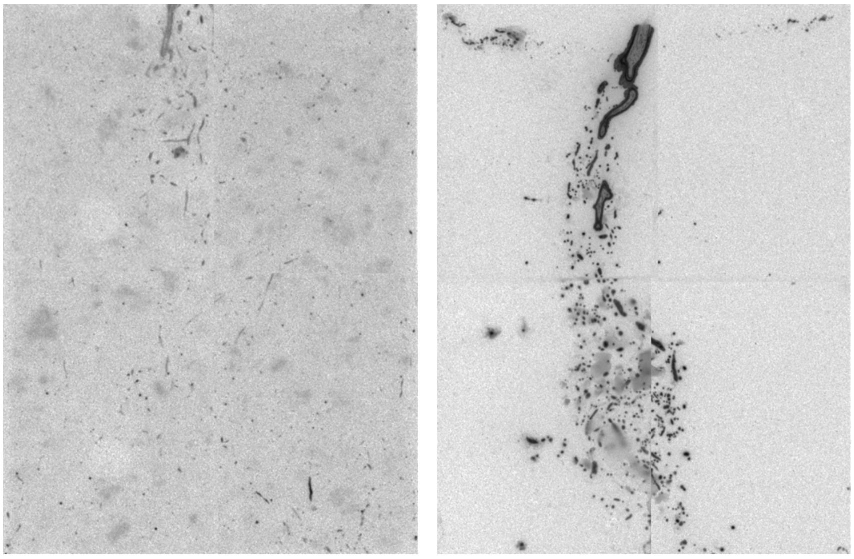

Figure 5: Example of an autoradiography of a soil sections from microcosms with *L. alpina* (left) and *P. mugo* (right). These sections are approximately 14 cm in vertical extent. Darker color indicates higher ^{14}C concentrations. The root system of *P. mugo* is well visible, with high concentrations in and at the surface of roots.
